# Uneven demographic consequences of the 2022 disease outbreak for the sea urchin *Diadema antillarum* in Puerto Rico

**DOI:** 10.7717/peerj.16675

**Published:** 2023-12-20

**Authors:** Ruber Rodríguez-Barreras, Claudia Patricia Ruiz-Diaz, Marcos A. Quiñones-Otero, Carlos Toledo-Hernández

**Affiliations:** 1 Department of Biology, University of Puerto Rico, Mayagüez campus, Mayagüez, Puerto Rico, USA; 2 Sociedad Ambiente Marino, San Juan, Puerto Rico, USA; 3 Río Piedras campus, Planning Department, University of Puerto Rico, San Juan, Puerto Rico, USA

**Keywords:** *Diadema antillarum*, Mass mortality, Sea urchin, Demography, Puerto Rico

## Abstract

Pervasive epizootic events have had a significant impact on marine invertebrates throughout the Caribbean, leading to severe population declines and consequential ecological implications. One such event was the regional collapse of herbivory, partly caused by the *Diadema antillarum* mortality event in 1983–84, resulting in a trophic cascade and altering the structure of reef communities. Consequently, there was a notable decrease in coral recruitment and an increase in the coverage of macroalgae. Nearly four decades later, in early 2022, the Caribbean basin experienced another widespread mass mortality event, further reducing the populations of *D. antillarum*. To assess the effects of this recent mortality event on the current demographics of *D. antillarum*, we surveyed eight populations along the eastern, northeastern, northern, and northwestern coast of Puerto Rico from May to July 2022, estimating their population density, size distribution, and disease prevalence. Additionally, the study compared these population parameters with data from four sites previously surveyed in 2012 and 2017 to understand the impact of the recent mortality event. The survey conducted in 2022 showed varying population densities at the surveyed reefs. Some populations exhibited mean densities of nearly one individual per square meter, while others had extremely low or no living individuals per square meter. The four populations with the highest density showed no evidence of disease, whereas the four populations with the lowest *D. antillarum* densities exhibited moderate to high disease prevalence. However, when considering all sites, the estimated disease prevalence remained below 5%. Nevertheless, the comparison with data from 2012 and 2017 indicated that the recent mortality event had a negative impact on *D. antillarum* demographics at multiple sites, as the densities in 2022 were reduced by 60.19% compared to those from the previous years. However, it is still too early to determine the severity of this new mortality event compared to the 1983–84 mortality event. Therefore, it is imperative to continue monitoring these populations.

## Introduction

Over recent years, the incidence of infectious diseases affecting marine organisms has increased and resulted in structural and functional impacts on ecosystems (Yadav & Upadhyay, 2023). Even though infectious diseases are common in the marine realm, mass mortalities caused by infectious diseases are rare, yet their effects can be dramatic and long-lasting. Mass mortalities could be particularly damaging in regions characterized by a low redundancy of functional groups, such as the Caribbean reefs (Carpenter, 1990; Mumby et al., 2006). Such events could lead to a functional extinction of a key species (*i.e.,* the level at which the species no longer fulfills its ecological role) (Valiente-Banuet et al., 2015), compromising the community assemblage of the entire region and consequently restructuring the services these ecosystems provide to humankind and their ecological roles in the oceans (Lessios, 1988; Carpenter, 1990).

The prevalence of disease outbreaks is increasingly impacting the marine environment (Harvell et al., 1999). Reports of large-scale episodic events leading to mass mortalities in marine organisms have increased since the latter half of the previous century (Hayes et al., 2001). Consequently, documented instances of population crash due to disease outbreak episodes have been reported in many marine taxa. However, population crashes are not uncommon among echinoderms (Hewson et al., 2019; Lawrence, 2020). This phylum is commonly referred to as a “boom-bust” group due to the frequent outbreak episodes and massive die-offs observed worldwide (Uthicke, Schaffelke & Byrne, 2009). Echinoderms play an important ecological role in the Caribbean region, not only as key herbivores, and structuring agents of the benthic community (Sammarco, 1982), but also due to their history of population crashes (Hughes et al., 1985; Steneck, 2013).

Several mass-mortality events affecting coral reefs in the Caribbean have been recorded, but the 1980 *Diadema antillarum* mass mortality event has been the most serious and well-studied of all (Lessios, 2016). Before the die-off, *D*. *antillarum* was among the main herbivores native to Caribbean coral reefs (Steneck, 2013; Mercado-Molina et al., 2015; Rodríguez-Barreras et al., 2014). However, a mysterious waterborne pathogen(s) demised over 98% of the *D. antillarum* population throughout the Caribbean basin (Lessios et al., 1984; Hughes et al., 1985). Immediately after, and in subsequent decades, reef-building corals declined, while the coral reefs experienced a significant increase in macroalgae, contributing to a severe decline in coral cover (Lessios, 2016). Nearly 40 years after the mass-mortality event, *D. antillarum* has shown variable levels of recovery across the region; however, *D. antillarum* densities have not reached pre-mortality levels in most localities (Mercado-Molina et al., 2015; Rodríguez-Barreras et al., 2015a; Tuohy, Wade & Weil, 2020; Pusack et al., 2022).

In early 2022, a new mortality event of *D. antillarum* was reported on several islands across the Caribbean (Response Network, AGRRA, 2022). The mortality was first reported in the US Virgin Islands, and subsequently, mortalities were reported in several reefs throughout the Caribbean. Many individuals have been found dead or showing signs of disease, *i.e.,* sea urchins outside their shelters in midday hours, unable to attach to the substrate, showing slow movement of spines as a response to contact, and loss of spines (Hylkema et al., 2023). It is known that the species has a diurnal sheltering and nocturnal foraging behavior (Sharp et al., 2023). The resurgence of the *D. antillarum* die-off at a time when populations across the Caribbean have not fully recovered is of great concern for the scientific community, given the poor ecological state of Caribbean coral reefs (Levitan, Best & Edmunds, 2023). Monitoring demographic changes in keystone species populations is essential for gaining insights into the biological relationships within an ecosystem. Therefore, in this study, we surveyed *D. antillarum* populations along the eastern, northern, and northwestern coasts of Puerto Rico. At each site, we estimated the population density, size distribution, and disease prevalence. We subsequently compared these parameters to available demographic data collected in 2012 and 2017 for four of these sites. These surveys were driven by three central questions: (1) Is the disease found in all the surveyed reefs? (2) Is the disease prevalence similar in all the surveyed reefs, and therefore are these reefs affected by the disease in a similar way, and (3) Are different sizes of *D.* *antillarum* individuals equally affected by the disease?

## Materials and Methods

### Surveyed sites

**Figure 1:**
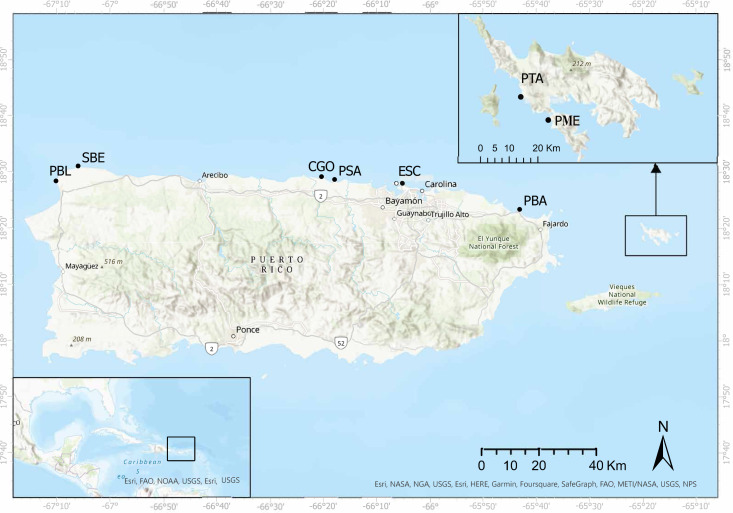
Surveyed sites along the eastern and northern coasts of Puerto Rico and Culebra Islands. Punta Tamarindo (PTA) and Punta Melones (PME) in Culebra Island, Punta Bandera (PBA), El Escambrón (ESC), Cerro Gordo (CGO), Playa Sardinera (PSA), Shacks Beach (SBE), and Playa Peña Blanca (PBL). Image credit: Open Street Map. Esri World Imaginary.

Surveys were carried out in eight shallow water reefs (<3.0 m depth) along the eastern, northeastern, northern, and northwestern coasts of Puerto Rico (Fig. 1). These sites were selected based on (1) the availability of demographic data from 2012 and 2017, and (2) divers who posted images of diseased and dead *D. antillarum* individuals on social media. The surveys started in May 2022, in Playa Punta Bandera located in Luquillo, on the northeastern coast (PBA), and Cerro Gordo in Vega Baja (CGO), on the northern coast of Puerto Rico. Surveys continued in June, 2022 when we visited Punta Tamarindo (PTA) and Punta Melones (PME), both on Culebra Island, on the eastern coast of Puerto Rico; Playa Sardinera in Dorado, on the northern coast (PSA), and Shacks Beach (SBE) and Playa Peña Blanca (PBL) in Aguadilla, both on the northwestern coast of Puerto Rico. Surveys ended in July 2022, when we visited Playa El Escambrón in San Juan on the northern coast (ESC) (Table 1).

**Table 1:** Surveyed sites along the eastern and northern coasts of Puerto Rico and Culebra Islands.

**Site**	**Acronym**	**County**	**Lat.**	**Long.**	**Coral cover (%)**	**Depth (m)**
Punta Tamarindo	PTA	Culebra	18.3151	−65.3179	15–20	1–2
Punta Melones	PME	Culebra	18.3041	−65.3112	15–20	1–3
El Escambrón	ESC	San Juan	18.4660	−66.0858	<5	0.5–3
Punta Bandera	PBA	Luquillo	18.3882	−65.7185	15–20	0.5–1
Cerro Gordo	CGO	Vega Alta	18.4850	−66.3389	<10	1–3
Shacks Beach	SBE	Isabela	18.5164	−67.1001	<5	1–3
Peña Blanca	PBL	Aguadilla	18.4724	−67.1691	<10	1–3
Playa Sardinera	PSA	Dorado	18.4768	−66.2984	<5	0.5–1

PBA is a shallow bordering reef (<2 m in depth), with coral cover ranging from 20–60% and dominated by standing dead and live *Acropora palmata* and *Pseudodiploria strigosa* at the reef crest. At the back reef, the substrate is dominated by *P. clivosa,* and *Porites furcata* mixed with patches of *Thalassia testudinum* and *Syringodium filiforme*. Water clarity is nearly 10 m year-round. CGO is a patchy reef interconnected with patches of seagrass beds dominated by *T. testudinum* and, to a lesser extent, by *S. filiforme*. This reef is influenced by a natural freshwater channel that drains nearly 100 m west of this reef. Coral cover at this reef is <10%, and is mainly dominated by *P. astreoides*, *P. strigosa*, and *P. clivosa*. Water clarity is highly variable, ranging from <2 m during the rainy season to >10 m in the dry season. PTA and PME are basaltic rock outcrops with coral coverage ranging from 15–20% dominated by massive coral heads such as *Porites astreoides*, *Pseudodiploria strigosa*, *P. clivosa*, *Favia fragum,* and standing dead *Acropora palmata*. Water clarity exceeds 10 m year-round. Data collected from these reefs were compared with historical data available in Rodríguez-Barreras et al. (2018).

PSA is a shallow (<1 m water depth) emergent aeolianite platform of 4.4 km^2^, bordered by seagrass beds dominated by *S. filiforme* and to a lesser extent *T. testudinum* and sand. Coral cover is nearly 8% and is dominated by *Madracis mirabilis*, *P. furcata*, and *Siderastrea radians*. Water clarity is highly variable, ranging from <1 m during the rainy season to >15 m in the dry season. SBE is dominated by dead coral heads of *Orbicella* and *Acropora*, mixed with *P. strigosa*, *P. clivosa*, and *A. palmata* at the reef crest and by *P. astreoides* and *A. palmata* and octocorals the back reef. In this zone, coral coverage is <5%. Water quality ranged between 3-10m most of the year. PBL is a karstic in origin flat substrate, with a coral cover <10%, dominated by *Pseudodiploria strigosa*, *P. clivosa*, *P. laberinthiformis*, *Porites astreoides* and octocorals. Water clarity is >8–10 m year-round. ESC is a submerged seawall of basaltic rocks and steel girders oriented perpendicular to the shore. Coral coverage at this reef is less than 5% and is dominated by *P. astreoides* and octocorals such as *Gorgonia ventalina*. The natural substratum next to the rocks is a mixed assemblage of macroalgae and sand with small size patches of *T. testudinum* and *S. filiforme*. Water clarity ranged between 1–5 m year-round.

### Population parameters

To determine sea urchin density, test diameter, and to be able to compare recently collected data with the historical data, we followed Mercado-Molina et al. (2015) and Rodríguez-Barreras et al. (2018). Briefly, at each reef and at hours ranging from 10:00–13:00, we set eight belt transects of 20 m^2^ (10 m × 2 m) parallel to the coast. Transects were at least 5 m apart from each other at depths ranging from 1–3 m, as at these depths sea urchin abundance tends to be higher (Ruiz-Ramos, Hernández-Delgado & Schizas, 2011; Rodríguez-Barreras et al., 2014; Mercado-Molina et al., 2015). We counted all individuals within each transect, including the healthy, the diseased, and the dead individuals. Sea urchin individuals were diagnosed as diseased if they were observed outside their cavities in daylight hours, unable to attach to the substrate, showing slow movement of spines as a response to contact and/or autotomy, *i.e.,* loss of spines. We also carefully inspected crevices between corals and small holes within each transect to avoid missing cryptic individuals. These data were used to estimate the urchin density (*i.e.,* the number of urchins per transect per site). We also measured the test diameter of individuals collected from the transects to estimate the size distribution at each reef. The total measured individuals per reef was 50. When needed, sea urchins out of the transects were measured until reaching 50 individuals per reef. Likewise, we also measured the tests from dead and sick sea urchins when possible. These data were used to classify sea urchins into three size classes: small or juvenile (test diameter ≤ 4.0 cm), medium or young adult (test diameter between 4.01 and 6.0 cm), and large or adult (test diameter >6.01 cm) individuals. This data was used to construct a size-frequency distribution (Miller et al., 2003; Lugo-Ascorbe, 2004; Rodríguez-Barreras et al., 2014). Sampling was approved by the Department of Natural and Environmental Resources of Puerto Rico, permit number DRNA- 2022-IC-046.

### Data analysis

We ran a general linear model with a Poisson distribution using the number of observations per transect as the response variable and the surveyed reefs as the explanatory variable to determine statistical differences between the 2022 sea urchin densities and between sites. To determine statistical significances between the historical density (*i.e.,* 2012 and 2017) and density data from 2022, we ran a general linear model with a Poisson distribution using the number of observed *D. antillarum* individuals per transect as the response variable and reefs (CGO, PTA, PME, and PBA) and years (*i.e.,* 2012, 2017 and 2022) as the explanatory variables. To compare size distribution based on the horizontal test diameter of *D. antillarum* among reefs during 2022, we used a two-way ANOVA, with the test size (in cm) as the response variable and size categories (small, medium, and large), and surveyed reefs as the explanatory variables. To determine potential differences in size structure from data collected in 2012, 2017, and 2022, we ran a three-way ANOVA using the test diameter (in cm) as the response variable and size categories (small, medium, and large), surveyed reefs (CGO, PTA, PME, and PBA) and years (2012, 2017 and 2022) as the explanatory variables, and a Tukey *post-hoc* pairwise comparison. All statistical analyses were conducted using R Statistical Software (v 4.3.1; R Core Team, 2023).

## Results

### Spatio-temporal abundance

**Figure 2:**
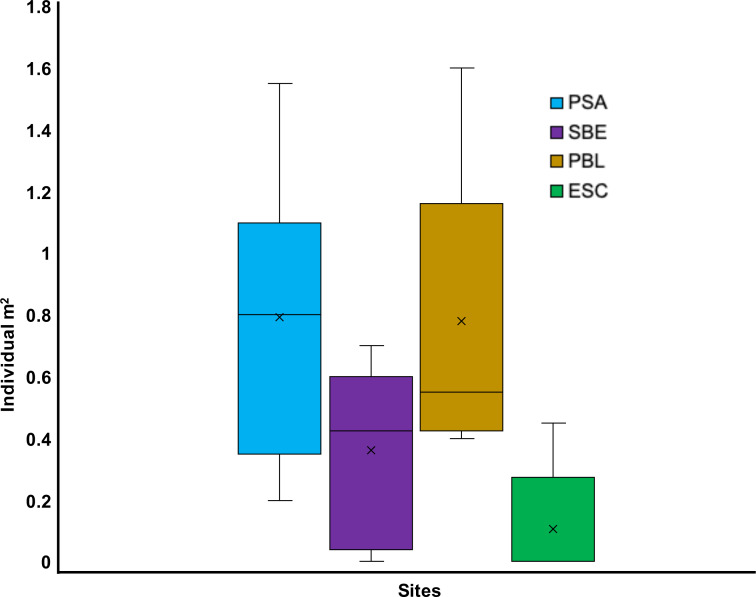
Boxplot showing the 2022 *Diadema antillarum* abundance across Cerro Gordo (CGO), El Escambrón (ESC), Punta Bandera (PBA), Playa Peña Blanca (PBL), Punta Melones (PME), Playa Sardinera (PSA), Punta Tamarindo (PTA), Shacks Beach (SBE). The red circle represents the mean, the median is represented by the bold line, the extremes of the boxplot are the 1st and 3rd quartiles, and the whiskers are the maximum and minimum. The black dots represent the outliers.

**Figure 3:**
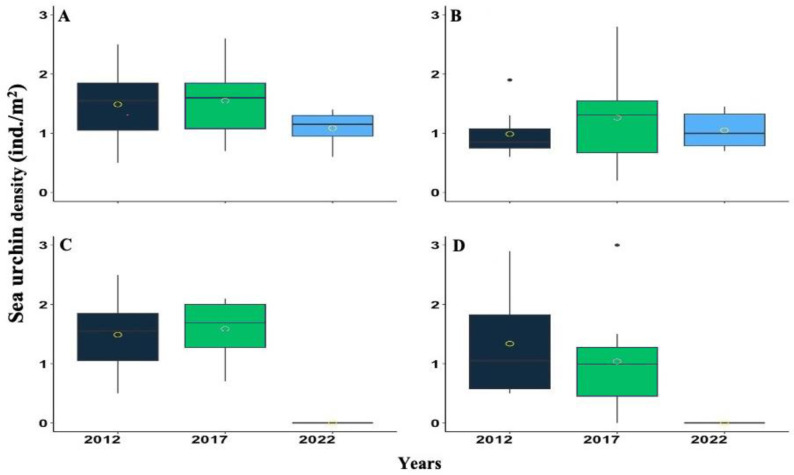
Boxplot showing the *Diadema antillarum* density across years 2012, 2017 & 2022 at (A) Cerro Gordo (CGO), (B) Punta Bandera (PBA), (C) Punta Tamarindo (PTA), and (D) Punta Melones (PME). In the boxplot, the yellow circle represents the mean, the median is presented by the bold line, the extreme of the boxplot are the 1st and 3rd quartiles, and the whiskers are the maximum and minimum.

Out of the eight sites visited from May to July 2022, seven had living *D. antillarum* (*i.e.,* PBA, CGO, PSA, SBE, PME, PBL, and ESC). We only observed dead individuals at PTA. Overall, a total of 665 living *D. antillarum* individuals were counted, resulting in a local mean density of 0.52 ±0.33 ind m^−2^ (mean ± SD). The highest densities were observed in CGO (1.09 ± 0.26 ind m^−2^) and PBA (1.05 ± 0.89 ind m^−2^), followed by PSA and PBL with 0.79 ± 0.43 ind m^−2^ and 0.78 ± 0.42 ind m^−2^, respectively. The lowest densities were found at PSA, ESC, and PME with 0.36 ± 0.27, 0.11 ± 0.17, and 0.01 ± 0.02 ind m^−2^, respectively (Figs. 2 and 3). The statistical analysis revealed significant differences in mean densities among all sites except between PBA and CGO (Table S1).

*D. antillarum* densities were highly variable from 2012, 2017, and 2022. Nonetheless, a consistent pattern of increasing from 2012 to 2017 and decreasing between 2017 to 2022 was observed at most of the surveyed reefs (Fig. 3). For instance, at CGO, density increased by 4.03% from 2012 to 2017, but from 2017 to 2022, it decreased by 29.84%. At PBA, density increased by 21.78% from 2012 to 2017 but decreased by 20.23% from 2017 to 2022 (Fig. 3). Likewise, at PTA, density increased by 6.30% from 2012 to 2017, yet no living individuals were observed in 2022. In contrast, density at PME steadily declined across the survey. For instance, from 2012 to 2017, density declined by 29%, and from 2017 to 2022 declined by 99%. Statistical differences were found among sites between 2012 and 2017 with 2022, and the interaction between sites and years (Table S2).

### Test diameter distribution

**Figure 4:**
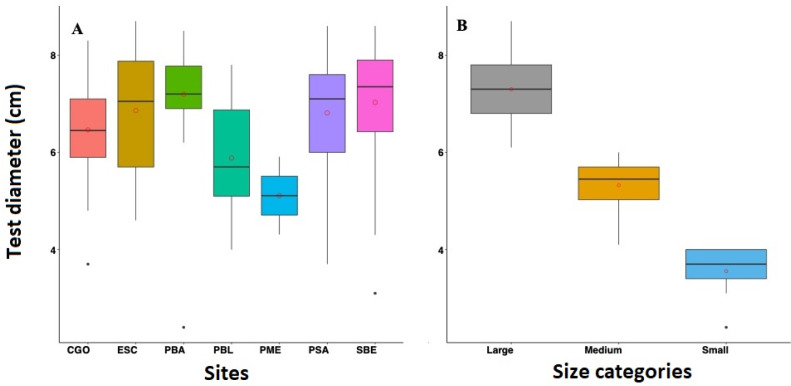
Boxplot showing the test diameter of *Diadema antillarum* in 2022 across the study sites (A) and by size categories (B). Sites are Cerro Gordo (CGO), Escambrón (ESC), Punta Bandera (PBA), Playa Peña Blanca (PBL), Punta Melones (PME), Playa Sardinera (PSA), and Shacks Beach (SBE). Size class category: small (≤ 4.0 cm), medium (4.01 to 6.01 cm), and large (>6.01 cm). In the boxplot, the red circle represents the mean, the median is presented by the bold line, the extreme of the boxplot are the 1st and 3rd quartiles, and the whiskers are the maximum and minimum.

**Figure 5:**
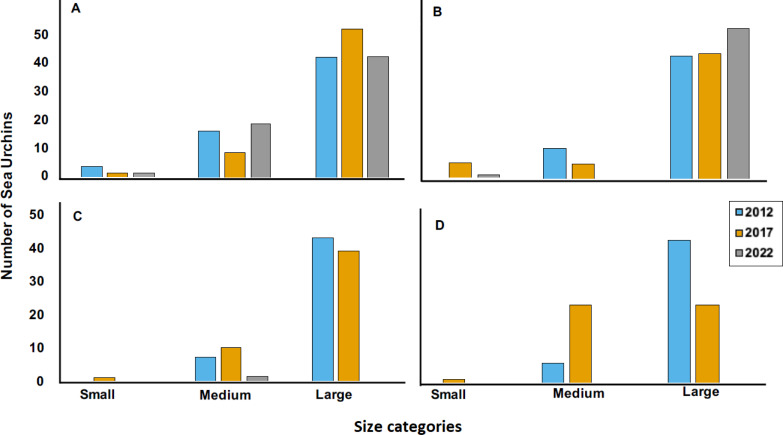
Size categories distribution across years 2012, 2017 and 2022 at four sites, where (A) is Cerro Gordo (CGO), (B) is Punta Bandera (PBA), (C) Punta Melones (PME), and (D) is Punta Tamarindo (PTA). Size class category: small (≤ 4.0 cm), medium (4.01 to 6.0 cm), and large (>6.01 cm).

Overall, the mean diameter of *D. antillarum* tests across reefs in 2022 were relatively similar. The highest test diameter was observed in PBA with 7.19 ± 0.89 cm, followed by SBE with 7.03 ± 1.33 cm, PSA with 6.81 ± 1.09 cm, ESC with 6.86 ± 1.23 cm, CGO with 6.46 ± 0.91 cm, and PBL with 5.84 ± 1.11 cm (Fig. 4). Only three individuals were measured at PME; the mean test diameter was 5.11 ± 1.07 cm. Similar test diameters were also recorded in 2012 and 2017. For instance, in 2012, PTA exhibited the highest mean test diameter at 6.82 ± 0.74 cm, followed by PME and PBA with 6.78 ± 0.77 cm and 6.75 ± 0.88 cm respectively, and lastly, CGO with 6.31 ± 1.31 cm. In 2017, CGO showed the highest test diameter with 6.79 ± 1.05 cm, followed by PBA and PME with 6.60 ± 1.13 cm and 6.57 ±1.10 cm, respectively, and lastly, PTA with 5.92 cm ±0.96 cm (Fig. 5). Statistical differences were detected between reefs (*F* = 5.334 *df* = 3, *p*-value = 0.001), years (*F* = 6.095, *df* = 2, *p*-value = 0.002), and the interaction between sites and years (*F* = 6.75, *df* = 5, *p*-value = 4.23 e-06). The post-hoc analysis revealed differences between PTA and the rest of the reefs across all years. The analysis also revealed differences between PBA-2022 and PME-2017, PBA-2017 and PTA-2017, PBA-2022, and CGO-2022.

The 2022 test size distribution was dominated by individuals from the large size class (individuals with tests >6.01 cm) in most reefs (Fig. 4). For instance, at PBA, 98% of the encountered individuals belong to the adult size class and only 2% to the small size class. At SBE, the large size class comprised 80% of the population, while the medium and small size classes represented 18% and 2%, respectively. Meanwhile, at CGO, PSA, and ESC, the large size class comprised between 70 to 76% of the population. Medium size class at ESC comprised 30%, while at PSA and CGO, the medium size class comprised 22 and 28%. The small size class individuals at CGO and PSA comprised around 2%. A similar demographic pattern was observed in the populations surveyed in 2012 (Fig. 5). For instance, the individuals from larger size class comprised between 80–88% of *D. antillarum* populations at PBA, CGO, PME, and PTA, whereas the medium size class (4.0 <*x* ≤ 6.01 cm) comprised between 12 to 20%. No small individuals (≤ 4.0 cm) were observed in 2012 at the surveyed sites, except in CGO (Fig. 5). However, by 2017 we observed a decrease in the larger individuals, *i.e.,* 86 to 50%, coupled with an increase in the medium size class, 10 to 50%, and the smaller size class, *e.g.*, 2 to 8%. The statistical analysis showed differences by reefs, with CGO showing statistical differences with PME and PTA. In addition, the analysis revealed significant differences by year, with 2012 being statistically different from 2022, and by size class categories, with the frequency of large individuals being different from medium and small individuals (Table 2).

**Table 2:** A post-hoc pairwise Tukey test comparison of horizontal test diameter among sites of Puerto Rico Island. Sites with no living Diadema (Punta Tamarindo (PTA) and Punta Melones (PME)) were excluded from the analysis. Sites are Punta Bandera (PBA), El Escambrón (ESC), Cerro Gordo (CGO), Playa Sardinera (PSA), Shacks Beach (SBE), and Playa Peña Blanca (PBL). Bolding indicates a significant difference.

**Sites**	CGO	PBA	DBE	PBL	SBE	ESC
CGO		**0.000**	0.054	**0.050**	**0.001**	0.110
PBA			0.083	**0.000**	0.714	0.243
PSA				**0.000**	0.171	0.885
PBL					**0.000**	**0.002**
SBE						0.373
ESC						

### Disease prevalence

**Figure 6:**
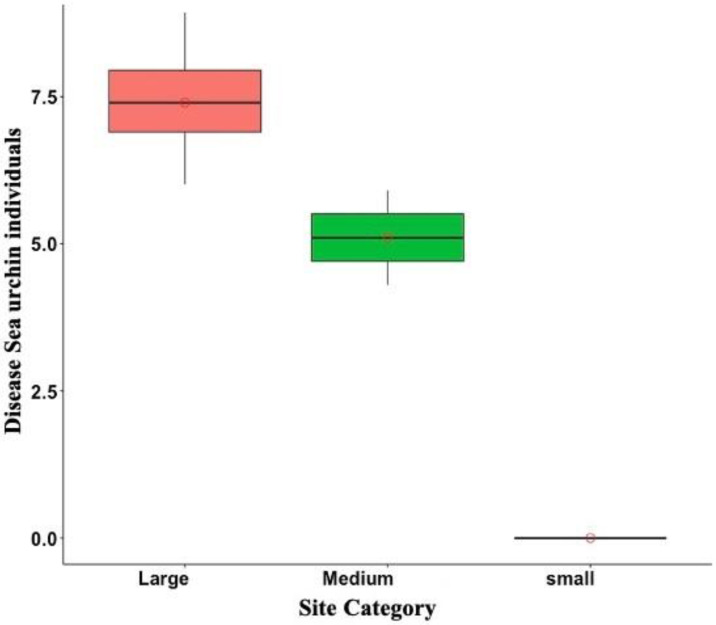
Overall disease sea urchin size class distribution in Puerto Rico for the eight surveyed sites in 2022. Notice that no small diseased individuals were observed. In the boxplot, the red circle represents the mean, the median is presented by the bold line, the extreme of the boxplot are the 1st and 3rd quartiles, and the whiskers are the maximum and minimum.

Of the total of 665 sea urchins counted from May to July of 2022, only 4.3% were diseased. Diseased sea urchins were exclusively observed in DBE, PME, PTA, and ESC, but disease prevalence varied among sites. For instance, disease prevalence at DBE and ESC was 11.02% and 41.17%, respectively. Meanwhile, the observed individuals at the Culebra sites were either diseased, as in the case of PME where two out of the three observed individuals were diseased, or, as in the case of PTA, there were no live individuals. In addition, among the diseased sea urchins, 92.6% of them belonged to the large size class, while the remaining 7% were accounted for medium size class. No small diseased individuals were observed during the surveys at any of the sites (Fig. 6).

## Discussion

### *D. antillarum* density

This study, conducted during the midst of the disease outbreak caused by *Scutico ciliatosis* (Hewson et al., 2023), focuses on assessing the current density and size distribution of *D. antillarum* in eight reefs located along the eastern, northeastern, northern, and northwestern coasts of Puerto Rico. Furthermore, to determine the impact of this die-off, the study compares the demographic parameters observed in 2022 with historical data. Our results indicate that the disease impact on *D. antillarum* populations was heterogeneous across all surveyed sites, with variations observed among different locations. However, all the observed diseased sea urchins exhibited external signs of illness similar to those described in the literature by Hylkema et al. (2023). Furthermore, the concurrent timing of our observations with those reported in other Caribbean jurisdictions strongly suggests that we were indeed dealing with the same disease.

Our data reveals significant variability in the estimated densities of *D. antillarum* across the surveyed reefs, with notable differences observed among sites. Of particular concern is the considerable decrease in density in 2022 compared to historical data for the Culebra sites. This reduction is significant considering that these sites had the highest densities on the eastern coast of Puerto Rico in the early 2000s (Ruiz-Ramos, Hernández-Delgado & Schizas, 2011). For instance, these authors reported mean densities of 1.59 ± 0.50 ind m^−2^ at PTA, whereas no living individuals were detected in 2022. We also documented similar density at PME, where density dropped from 1.04 ± 0.90 ind m^−2^ in 2017 to no healthy sea urchins detected in 2022. Other sites have experienced similar *D. antillarum* reductions. For instance, Rodríguez-Barreras et al. (2014) reported densities of 1.10 ind m^−2^ at ESC, yet our 2022 survey revealed a density drop to 0.1 ind m^−2^, with nearly half of the individuals being affected by the disease. Given the current disease prevalence observed at ESC, it is anticipated that the outbreak will have a more significant impact if the diseased individuals do not recover, and the disease continues to spread. The other sites exhibited less severe outbreak impact. For instance, PSA and PBL exhibited similar densities, but PSA displayed a disease prevalence of over 10%, while no diseased individuals were detected in PBL. Therefore, the density at PSA would likely have been higher than that of PBL if it had not been affected by the outbreak event.

Densities at PBA and CGO have also experienced decreases even though evidence of disease was not found. For instance, a study conducted in 2017 by (Rodríguez-Barreras et al., 2018) estimated the density at PBA at 1.26 ind m^−2^, while the estimated current density decreased to 1.05 ind m^−2^. A similar declining trend was observed in CGO, where densities decreased from 1.55 ind m^−2^ in 2017 to 1.09 ind m^−2^ in 2022. respectively. In subsequent visits to these sites in February, May, and August 2023, we found no evidence of the disease, further suggesting that the disease may not be the primary cause of these declines (unpublished data). Instead, habitat degradation may have influenced the observed declines as multiple coral heads, including the dominant species in this area *e.g.*, *Pseudodiploria* spp., were either recently dead or exhibited signs of Stony Coral Tissue Loss Disease (Dahlgren et al., 2021). Alternatively, the absence of diseased or dead sea urchins among the studied reefs may result in some populations being more resistant to the disease than others. It is also noteworthy to mention that from 2017 to 2022, three hurricanes onslaught Puerto Rico. These hurricanes caused significant damage to the reefs and seagrass beds, which, when combined with natural low recruitment, may have resulted in the observed low density in 2022 when compared to the 2017 densities (Miller et al., 2009; Rodríguez-Barreras et al., 2015a; Rodríguez-Barreras et al., 2015b; Pilnick et al., 2021).

### Size distribution

The current size distribution of *D. antillarum* in the surveyed reefs was dominated by the large-size class individuals, with fewer medium-sized class individuals and even fewer small-size class individuals. The absence of juveniles may have multiple explanations. For instance, lower frequencies of small-size individuals may suggest a generally low recruitment given the relatively low abundance of mature and, therefore, larger *D. antillarum* individuals when compared to 80s pre-mortality events. In fact, most authors argued this as the main reason for the slow recovery after the massive mortality (Lessios, 1988; Miller et al., 2003; Rodríguez-Barreras et al., 2018). It could also suggest a high mortality among the recently settled and juvenile sea urchins due to predation, as several studies have argued that *D. antillarum* predation by reef-fishes may have a profound effect on the sea urchin size structure (Harborne et al., 2009; Rodríguez-Barreras et al., 2015a). Nonetheless, it is difficult to conclude that the reason for the low observations of juveniles and sub-adults was related to being more susceptible to the disease than mature individuals. Therefore, longitudinal studies, which include a collection of *D. antillarum* in the larval pool and recruit monitoring across several reefs, are required to better comprehend the demographic dynamics of *D. antillarum* under the current outbreak scenario.

### Outbreak impact

Overall, the prevalence of the new outbreak was still relatively low in the surveyed reefs, as only 4.3% of the 665 counted sea urchins were diseased (Fig. 6). It also shows an erratic geographic distribution, with some reefs showing high prevalence and others with moderately to low prevalence and hence presumably low impact. A recent study conducted in the Dominican Republic also reported variable impact across different reefs, although the outbreak in the Dominican Republic seems to have had a higher impact than in Puerto Rico (Villalpando et al., 2022). Nonetheless, the outbreak in Puerto Rico just started, as judged by the lack of conclusive evidence of disease in some of the surveyed reefs. Therefore, it is still premature to capture the real magnitude of the impact, and if the ongoing outbreak is as destructive as the 80s mortality event when populations were decimated throughout the western Atlantic, including the Bermudas, in a relatively short period of time (Mumby et al., 2006; Bove, Mudge & Bruno, 2022). Instead, the spatial-heterogeneous nature of this new outbreak and the variable mortality of individuals resemble the mortality event occurring in the sibling urchin species *D. africanum*, from October 2009 to April 2010 in the subtropical eastern Atlantic (Clemente et al., 2014). Nonetheless, long-term monitoring programs at these reefs may help disclose size class patterns of disease susceptibility.

## Conclusion

Populations of *D. antillarum* at surveyed reefs have not fully recovered since the mass mortality event in the 1980s (Mercado-Molina et al., 2015; Rodríguez-Barreras et al., 2018) and are now facing a second outbreak that is causing further damage. This study sheds light on the current state of *D. antillarum* populations in Puerto Rico’s reefs amid the *Scutico ciliatosis* outbreak, revealing different degrees of impact across different locations, with some reefs experiencing a drastic decline in sea urchin density, particularly concerning for sites like Culebra, which once boasted relative high densities. Nonetheless, the limited available data makes it difficult to determine which factors (abiotic and biotic) may favor the infection either by compromising the individual’s immune system or by favoring the proliferation of the biotic agent(s) or both. This 2022 outbreak’s complexity mirrors past events, emphasizing the importance of establishing long-term monitoring programs where key abiotic and biotic components known to cause stress to other coral reefs-associated organisms are regularly surveyed. This is especially critical in the face of climate change and changing marine conditions, which may weaken the immunity of marine organisms and increase the frequency and severity of disease outbreaks.

##  Supplemental Information

10.7717/peerj.16675/supp-1 Supplemental Information 1 Comparison of sea urchin abundance between sites Cerro Gordo (GGO) and El Escambrón (ESC), Punta Bandera (PBA), Playa Peña Blanca (PBL), Punta Melones (PME), Playa Sardinera (PSA), Punta Tamarindo (PTA), and Shacks Beach (SBE) over 2022 using a General Linear Model with Poison distribution, AIC= 655.75. The asterisks (*) indicate the level of significance.

10.7717/peerj.16675/supp-2 Supplemental Information 2 Comparison of sea urchin abundance between Cerro Gordo (GGO) and Punta Bandera (PBA), Punta Melones (PME), and Punta Tamarindo (PTA) over the years 2012, 2017, and 2022 using a General Linear Model with Poison distribution, AIC=655.75 The asterisks indicate the level of significance. Note: no sea urchin was observed in 2022.

10.7717/peerj.16675/supp-3 Supplemental Information 3 Raw data (abundance and test size diameter) The number of sea urchins found at each transect and the test diameter of 50 individuals (when possible) for 8 sites in Puerto Rico.

## Additional Information and Declarations

### Data Availability

The following information was supplied regarding data availability: The raw data is available in the Supplementary File.

## References

[ref-1] AGRRA (2022). Diadema response network map of diadema and other sea urchins in the Caribbean. ArcGIS Online. https://www.agrra.org/sea-urchin-die-off/.

[ref-2] Bove CB, Mudge L, Bruno JF (2022). A century of warming on Caribbean reefs. PLOS Climate.

[ref-3] Carpenter RC (1990). Mass mortality of *Diadema antillarum*. Marine Biology.

[ref-4] Clemente S, Lorenzo-Morales J, Mendoza JC, López C, Sangil C, Alves F, Kaufmann M, Hernández JC (2014). Sea urchin *Diadema africanum* mass mortality in the subtropical eastern Atlantic: role of waterborne bacteria in a warming ocean. Marine Ecology Progress Series.

[ref-5] Dahlgren C, Pizarro V, Sherman K, Greene W, Oliver J (2021). Spatial and temporal patterns of stony coral tissue loss disease outbreaks in the Bahamas. Frontiers in Marine Science.

[ref-6] Harborne AR, Renaud PG, Tyler EHM, Mumby PJ (2009). Reduced density of the herbivorous urchin Diadema antillarum inside a Caribbean marine reserve linked to increased predation pressure by fishes. Coral Reefs.

[ref-7] Harvell CD, Kim K, Burkholder JM, Colwell RR, Epstein PR, Grimes DJ, Hoffman EE, Lipp EK, Osterhaus DME, Overstreet RM, Porter JW, Smith GW, Vasta GR (1999). Emerging marine diseases–climate links and anthropogenic factors. Science.

[ref-8] Hayes ML, Bonaventura J, Mitchell TP, Prospero JM, Shinn EA, Van Dolah F, Barber RT (2001). How are climate and marine biological outbreaks functionally linked?. The Ecology and Etiology of Newly Emerging Marine Diseases.

[ref-9] Hewson I, Ritchie IT, Evans JS, Altera A, Behringer D, Bowman E, Brandt M, Budd KA, Camacho RA, Cornwell TO, Countway PD, Croquer A, Delgado GA, Derito C, Duermit-Moreau E, Francis-Floyd R, Henderson L, Hylkema A, Kellogg CA, Kiryu Y, Kitson-Walters KA, Kramer P, Lang JC, Lessios H, Liddy L, Marancick D, Nimrod S, Patterson JT, Pistor M, Romero IC, Sellares-Blasco R, Servier MLB, Sharp WC, Souza M, Valdez-Trinidad A, Van der Lann M, Villanova-Cuevas B, Villalpando M, Von Hoene SH, Warham M, Wijers T, Williams SM, Work TM, Zambrano S, Zimmermann A, Breitbart MYA (2023). A scuticociliate causes mass mortality of *Diadema antillarum* in the Caribbean Sea. Science Advances.

[ref-10] Hewson I, Sullivan B, Jackson EW, Xu Q, Long H, Lin C, Quijano-Carde EV, Seymour J, Siboni N, Jones MRL, Sewell MA (2019). Perspective: something old, something new? Review of wasting and other mortality in Asteroidea (Echinodermata). Frontiers in Marine Science.

[ref-11] Hughes TP, Keller BD, Jackson JBC, Boyle MJ (1985). Mass mortality of the echinoid *Diadema antillarum* Philippi in Jamaica. Bulletin of Marine Science.

[ref-12] Hylkema A, Kitson-Walters K, Kramer PR, Patterson JT, Roth L, Sevier ML, Vega-Rodriguez M, Warham MM, Williams SM, Lang JC (2023). The 2022 *Diadema antillarum* die-off event: comparisons with the 1983–1984 mass mortality. Frontiers in Marine Science.

[ref-13] Lawrence JM (2020). Mass mortality of echinoderms from abiotic factors. Echinoderm studies 5 1996.

[ref-14] Lessios HA (1988). Mass mortality of *Diadema antillarum* in the Caribbean: what have we learned?. Annual Review of Ecology and Systematics.

[ref-15] Lessios HA (2016). The great *Diadema antillarum* die-off: 30 years later. Annual Review of Marine Science.

[ref-16] Lessios HA, Cubit JD, Robertson DR, Shulman MJ, Parker MR, Garrity SD, Levings SC (1984). Coral Reefs.

[ref-17] Levitan DR, Best RM, Edmunds PJ (2023). Sea urchin mass mortalities 40 y apart further threaten Caribbean coral reefs. Proceedings of the National Academy of Sciences of the United States of America.

[ref-18] Lugo-Ascorbe MA (2004). Population status of the black sea urchin *Diadema antillarum* (Philippi) in La Parguera, Puerto Rico, 20 years after the mass mortality event. Doctoral dissertation.

[ref-19] Mercado-Molina AE, Montañez Acuña A, Rodríguez-Barreras R, Colón-Miranda R, Díaz-Ortega G, Martínez-González N, Schleier-Hernández Sandra, Sabat AM (2015). Revisiting the population status of the sea urchin *Diadema antillarum* in northern Puerto Rico. Journal of the Marine Biological Association of the United Kingdom.

[ref-20] Miller MW, Kramer KL, Williams SM, Johnston L, Szmant AM (2009). Assessment of current rates of *Diadema antillarum* larval settlement. Coral Reefs.

[ref-21] Miller RJ, Adams AJ, Ogden NB, Ogden JC, Ebersole JP (2003). *Diadema antillarum* 17 years after mass mortality: is recovery beginning on St. Croix?. Coral Reefs.

[ref-22] Mumby PJ, Hedley JD, Zychaluk K, Harborne AR, Blackwell PG (2006). Revisiting the catastrophic die-off of the urchin *Diadema antillarum* on Caribbean coral reefs: fresh insights on resilience from a simulation model. Ecological Modelling.

[ref-23] Pilnick AR, O’Neil KL, Moe M, Patterson JT (2021). A novel system for intensive *Diadema antillarum* propagation as a step towards population enhancement. Scientific Reports.

[ref-24] Pusack TJ, Stallings CD, Albins MA, Benkwitt CE, Ingeman KE, Kindinger TL, Hixon MA (2022). Protracted recovery of long-spined urchin (*Diadema antillarum*) in the Bahamas. Coral Reefs.

[ref-25] R Core Team (2023). https://www.R-project.org/.

[ref-26] Rodríguez-Barreras R, Durán A, Lopéz-Morell J, Sabat AM (2015b). Effect of fish removal on the abundance and size structure of the sea urchin *Diadema antillarum*: a field experiment. Marine Biology Research.

[ref-27] Rodríguez-Barreras R, Montanez-Acuna A, Otano-Cruz A, Ling SD (2018). Apparent stability of a low-density *Diadema antillarum* regime for Puerto Rican coral reefs. ICES Journal of Marine Science.

[ref-28] Rodríguez-Barreras R, Pérez ME, Mercado-Molina AE, Sabat AM (2015a). Arrested recovery of *Diadema antillarum* population: survival or recruitment limitation?. Estuarine, Coastal and Shelf Science.

[ref-29] Rodríguez-Barreras R, Pérez ME, Mercado-Molina AE, Williams SM, Sabat AM (2014). Higher population densities of the sea urchin *Diadema antillarum* linked to wave sheltered areas in north Puerto Rico Archipelago. Journal of the Marine Biological Association of the United Kingdom.

[ref-30] Ruiz-Ramos DV, Hernández-Delgado EA, Schizas NV (2011). Population status of the long-spined urchin *Diadema antillarum* in Puerto Rico 20 years after a mass mortality event. Bulletin of Marine Science.

[ref-31] Sammarco PW (1982). Polyp bail-out: an escape response to environmental stress and a new means of reproduction in corals. Marine Ecology Progress Series. Oldendorf.

[ref-32] Sharp WC, Delgado GA, Pilnick AR, Patterson JT (2023). Diurnal sheltering behavior of hatchery-propagated long-spined urchins (*Diadema antillarum*): a re-examination following husbandry refinements. Bulletin of Marine Science.

[ref-33] Steneck RS (2013). Sea urchins as drivers of shallow benthic marine community structure. Developments in Aquaculture and Fisheries Science.

[ref-34] Tuohy E, Wade C, Weil E (2020). Lack of recovery of the long-spined sea urchin *Diadema antillarum* Philippi in Puerto Rico 30 years after the Caribbean-wide mass mortality. PeerJ.

[ref-35] Uthicke S, Schaffelke B, Byrne M (2009). A boom–bust phylum? Ecological and evolutionary consequences of density variations in echinoderms. Ecological Monographs.

[ref-36] Valiente-Banuet A, Aizen MA, Alcántara JM, Arroyo J, Cocucci A, Galetti M, García María B., García Daniel, Gómez José M., Jordano Pedro, Medel Rodrigo, Navarro Luis, Obeso José R., Oviedo Ramona, Ramírez Nelson, Rey PJ, Traveset Anna, Verdú Miguel, Zamora Regino, Johnson Marc (2015). Beyond species loss: the extinction of ecological interactions in a changing world. Functional Ecology.

[ref-37] Villalpando MF, Guendulain-García SD, Valdez-Trinidad A, Croquer A, Sellares-Blasco RI (2022). Coral reefs of southeastern Dominican Republic hit by two simultaneous epizootic events. Bulletin of Marine Science.

[ref-38] Yadav N, Upadhyay RK (2023). Global effect of climate change on seasonal cycles, vector population and rising challenges of communicable diseases: a review. Journal of Atmospheric Science Research.

