# Supplementary material for: Uneven demographic consequences of the 2022 disease outbreak for the sea urchin Diadema antillarum in Puerto Rico
Source: PeerJ. 2023 Dec 20;11:e16675. doi: 10.7717/peerj.16675 (PMC10748467; doi:10.7717/peerj.16675)
Supplement: Supplemental Information 1 — Cerro Gordo (GGO) and El Escambrón (ESC), Punta Bandera (PBA), Playa Peña Blanca (PBL), Punta Melones (PME), Playa Sardinera (PSA), Punta Tamarindo (PTA), and Shacks Beach (SBE) over 2022 using a General Linear Model with Poison distribution, AIC= 655.75. The asterisks (*) indicate the level of significance. [file peerj-11-16675-s001.docx]

**Supp. Table S1**. Comparison of sea urchin abundance between sites: Cerro Gordo (GGO) and El Escambrón (ESC), Punta Bandera (PBA), Playa Peña Blanca (PBL), Punta Melones (PME), Playa Sardinera (PSA), Punta Tamarindo (PTA), and Shacks Beach (SBE) over 2022 using a General Linear Model with Poison distribution, AIC= 655.75. The asterisks (*) indicate the level of significance.

| **Sites** | **Estimate** | **Std Error** | **z value** | **Pr (>\|z\|)** | |
| --- | --- | --- | --- | --- | --- |
| Intercept | 3.07961 | 0.07581 | 40.623 | < 2.00E-16 | *** |
| **ESC** | -2.32584 | 0.25411 | -9.153 | < 2.00E-16 | *** |
| **PBA** | -0.03509 | 0.10816 | -0.324 | 0.74562 |  |
| **PBL** | -0.33074 | 0.11725 | -2.821 | 0.00479 | ** |
| **PME** | -4.06044 | 0.58231 | -6.973 | 3.10E-12 | *** |
| **PSA** | -0.31487 | 0.11671 | -2.698 | 0.00698 | ** |
| **PTA** | -3.21315 | 0.38541 | -8.337 | < 2.00E-16 | *** |
| **SBE** | -1.09861 | 0.15162 | -7.246 | 4.30E-13 | *** |
