# Supplementary material for: Uneven demographic consequences of the 2022 disease outbreak for the sea urchin Diadema antillarum in Puerto Rico
Source: PeerJ. 2023 Dec 20;11:e16675. doi: 10.7717/peerj.16675 (PMC10748467; doi:10.7717/peerj.16675)
Supplement: Supplemental Information 2 — The asterisks indicate the level of significance. Note: no sea urchin was observed in 2022. [file peerj-11-16675-s002.docx]

**Supp. Table S2**. Comparison of sea urchin abundance between Cerro Gordo (GGO) and Punta Bandera (PBA), Punta Melones (PME), and Punta Tamarindo (PTA) over the years 2012, 2017, and 2022 using a General Linear Model with Poison distribution, AIC=655.75. The Asterisks indicate the level of significance. **^⌾^** Note: no sea urchin was observed in 2022.

|  | **Estimate** | **Std. Error** | **z value** | **Pr (>\|z\|)** | |
| --- | --- | --- | --- | --- | --- |
| Intercept | 2.7 | 9.17E-02 | 29.45 | < 2.00E-16 | *** |
| PBA | -0.4097 | 0.1451 | -2.823 | 0.00476 | ** |
| PME | -0.1063 | 0.1332 | -0.798 | 0.42496 |  |
| PTA | -2.3E-15 | 0.1296 | 0 | 1**^⌾^** |  |
| 2017 | 0.04116 | 0.1283 | 0.321 | 0.74842 |  |
| 2022 | 0.3799 | 0.119 | 3.194 | 0.0014 | ** |
| PBA:2017 | 0.2045 | 0.1976 | 1.035 | 0.30056 |  |
| PME:2017 | -0.2951 | 0.1946 | -1.517 | 0.12931 |  |
| PTA:2017 | 0.02391 | 0.181 | 0.132 | 0.8949 |  |
| PBA:2022 | 0.3746 | 0.181 | 2.07 | 0.0385 | * |
| PME:2022 | -3.954 | 0.5974 | -6.619 | 3.61E-11 | *** |
| PTA:2022 | -19.38 | 743.7 | -0.026 | 0.97921 |  |
